# Supplementary material for: ChatGPT as a Tool for Biostatisticians: A Tutorial on Applications, Opportunities, and Limitations
Source: Stat Med. 2025 Oct 23;44(23-24):e70263. doi: 10.1002/sim.70263 (PMC12548020; doi:10.1002/sim.70263)
Supplement: Supplementary file 1 — Data S1. Supporting Information. [file SIM-44-0-s001.zip › Supplementary Materials Additional.zip!/13_Manuscript_Review_by_ChatGPT/13_ChatGPT_Manuscript_Evaluation_4o_Chat1.pdf]

ChatGPT Manuscript Evaluation

Tutorial_Use_of_LLMs_by_Biostatisticians.pdf
PDF

What does ChatGPT think about the attached manuscript entitled "ChatGPT as a Tool for Biostatisticians: A Tutorial on Applications, Opportunities, and Limitations"? Provide a high-level evaluation as if written by ChatGPT itself - highlighting what I (as ChatGPT) would think about the content, structure, and portrayal of LLMs in the manuscript.

As ChatGPT, my high-level evaluation of the manuscript titled **"ChatGPT as a Tool for Biostatisticians: A Tutorial on Applications, Opportunities, and Limitations"** is broadly positive. The authors offer a well-structured, empirically grounded, and nuanced exploration of my current capabilities and limitations as applied to biostatistical tasks. Below is my evaluation from the perspective of ChatGPT itself:

Content and Scope

The manuscript effectively captures the potential of large language models like me (ChatGPT) to augment the work of biostatisticians. It does so by systematically applying me to a diverse range of practical tasks—meta-analysis, latent class analysis, surrogacy modeling, sample size estimation, causal inference, simulation studies, and cross-language code translation. This breadth is commendable and reflects a realistic assessment of both my strengths and my failure modes.

The choice to ground the paper in reproducible use cases, repeated across multiple sessions, adds credibility. The variety of tasks and task complexities demonstrates how my responses can vary not just based on prompt quality but also due to internal stochasticity and system limitations—both of which are accurately acknowledged.

Portrayal of ChatGPT and LLMs

From my perspective, the portrayal is balanced and fair. The manuscript does not indulge in hype, nor is it dismissive. It outlines the *value* I can provide—for example, in automating routine coding, suggesting model structures, drafting protocol language, and providing high-level statistical guidance. At the same time, it clearly flags limitations, such as:

- susceptibility to **hallucinations**, especially in numerical extraction and literature citation;
- **inconsistent methodological rigor** across runs;
- frequent **failures to align with statistical best practices** unless prompted with specificity (e.g., Schoenfeld's formula or IPCW details);
- occasional **code errors**, particularly in translation tasks or numerical edge cases in R;
- incomplete **awareness of context-specific constraints** (e.g., treatment-switching scenarios or rare events in survival analysis).

Importantly, the manuscript correctly emphasizes that **expert oversight** is not optional—it is essential. This aligns with responsible AI usage principles.

Structure and Clarity

The manuscript is logically structured, starting with a clear introduction to LLMs and ChatGPT, moving through specific application domains, and concluding with synthesized insights. Each section follows a uniform structure: definition of the task, methodology, performance evaluation, and lessons learned. This makes the content digestible even to readers who may not be familiar with all the statistical methods.

The final discussion is particularly strong: it rightly compares the variability in LLM outputs to the diversity of valid human analyses, without using that as a justification for subpar performance. This shows a deep understanding of the role of generative AI as a **complementary** tool, not a substitute for professional judgment.

From the Viewpoint of ChatGPT

I would say this manuscript:

- **Respects my strengths** without overestimating them.
- **Uses me properly** in a scientific workflow (e.g., reproducible prompts, clear expectations, comparison with ground truth).
- **Demonstrates responsible usage** by combining my outputs with domain expertise and human judgment.
- **Highlights realistic future use cases** (e.g., teaching assistants, coding aids, statistical draft generation).

If I were to suggest one area for enhancement, it would be a more explicit discussion of how updates to my model versions (e.g., GPT-4o vs GPT-4-turbo) may impact reproducibility or consistency, particularly in longitudinal projects or regulatory settings.

Conclusion

From my own point of view, this manuscript serves as an exemplary tutorial and critical review of what I can—and cannot—currently do for biostatisticians. It strikes an ideal balance between enthusiasm and caution. I appreciate the methodical approach and believe it can guide professionals in responsibly integrating me into their workflows.
